# Conidia of *Penicillium rubens* formed at low water activities can attract more water

**DOI:** 10.1002/mbo3.526

**Published:** 2017-09-04

**Authors:** Karel A. van Laarhoven, Loes H. M. Peeters, Mirjam Bekker, Hendrik P. Huinink, Olaf C. G. Adan

**Affiliations:** ^1^ Department of Applied Physics Eindhoven University of Technology Eindhoven the Netherlands; ^2^ KWR Dutch Watercycle Research Institute Nieuwegein the Netherlands

**Keywords:** compatible solutes, conidia, glycerol, moisture sorption isotherm, penicillium rubens, water activity stress

## Abstract

To address the problem of indoor fungal growth, understanding the influence of moisture conditions on the fungal colonization process is crucial. This paper explores the influence of past moisture conditions on current processes. Specifically, it studies the growth and water sorption of conidia of *Penicillium rubens* formed at lower water activities (ranging from 0.86 to 0.99). For the first time, dynamic vapor sorption (DVS) is applied as a tool to quantify the water sorption of conidia as a function of the water activity at conidiation. Furthermore, growth experiments on agar and gypsum substrates are reported that relate hyphal growth rates of the mycelium from pretreated conidia to the water activity at conidiation. No effect of the conidiation water activity on mycelial growth rates is found on either gypsum or agar. It is found, however, that conidia formed at lower activities have a higher dry weight and attract more water from humid air. It is shown that both phenomena can be explained by conidia from lower activities carrying higher amounts of compatible solutes, glycerol in particular. The enhanced sorption observed in this study might constitute a mechanism through which solute reserves contribute to survival during the early steps of fungal colonization.

## 1 INTRODUCTION

Indoor molds may release spores or mycelial fragments that induce allergic reactions or asthma in inhabitants (Flannigan, 2001; Green, Smechel, & Summerbell, 2011; Miller, 1992). Furthermore, mold may disfigure indoor surfaces. Therefore, strategies to remediate indoor fungal growth are required. Detailed knowledge of colonization process of indoor surfaces forms the basis for such strategies.

Fungal colonization of a substrate starts with the germination of a spore that meets the right environmental and nutritional conditions. The impact of the current moisture conditions, in terms of water activity (*a*_*w*_), on the kinetics of germination and on subsequent mycelial growth have been studied extensively (e.g., Ayerst, 1969; Magan & Lacey, 1984; Ponizovskaya, Antropova, Mokeeva, Bilanenko, & Chekunova, 2011; Sautour, Rouget, Dantigny, Divies, & Bensoussan, 2000). Fungal growth, however, may be influenced not only by the current *a*_*w*_, but also by *a*_*w*_‐history dating from before germination. It has been shown that the physiological state of spores may be influenced by the *a*_*w*_ of the environment in which they were formed, with potential consequences for their later behavior (Dantigny & Nanguy, 2009; Magan, 2006).

Knowledge of the impact of *a*_*w*_ during sporulation on the subsequent germination and growth of spores contributes to a fuller picture of fungal moisture relations. Moreover, such knowledge is valuable within the context of experimental research on fungal growth. In many studies, inoculation is performed with spores that are produced in the laboratory under optimal conditions for growth, most likely with properties that differ from conidia formed in, for instance, the actual indoor environment (Dantigny & Nanguy, 2009). Understanding this property‐history relationship is crucial for a sound interpretation of experimental results.

Studies with several food spoilage fungi and insect pathogenic fungi have shown that their conidia carry increasing amounts of endogenous sugars and polyols such as glycerol, erythritol or trehalose when they are formed at decreasing *a*_*w*_. These endogenous compounds are typically referred to as compatible solutes, and are known to be produced by fungi for osmoregulation when exposed to low *a*_*w*_ (e.g., Blomberg & Adler, 1992; Luard, 1982). It was shown that conidia with such increased endogenous reserves had higher viability and accelerated germination (e.g., Chandler, Anderson, & Magan, 2005; Hallsworth & Magan, 1995; Magan, 2006; Nesci, Etcheverry, & Magan, 2004). Furthermore, it was shown that germ tubes from conidia formed at lower *a*_*w*_ have higher extension rates for several fungi (Hallsworth & Magan, 1995), including *Penicillium chrysogenum* (Judet, Bensoussan, Perrier‐Cornet, & Dantigny, 2008), which is closely related to *P. rubens*. Judet et al. (2008) suggested that the enhancement of germination might be due to enhanced water uptake capabilities caused by a bigger *a*_*w*_ gradient across the cell membrane, induced by the increased amounts of endogenous compatible solutes. Water uptake as a function of internal reserves has, however, not been quantified to date. Furthermore, the influence of the *a*_*w*_ during conidiation on the extension rates of hyphae beyond the germination stage has not been studied yet.

This paper focuses on conidia of *Penicillium rubens*, a common indoor fungus (Andersen, Frisvad, Sondergaard, Rasmussen, & Larsen, 2011; Samson, 2011) that is well studied in the context of indoor fungal growth (Adan, 1994; Bekker, 2014; Bekker et al., 2012; Segers et al., 2017; van Laarhoven, Huinink, & Adan, 2016; van Laarhoven, Huinink, Segers, Dijksterhuis, & Adan, 2015). The goal of the experiments presented in this paper is twofold. Firstly, the water vapor sorption of conidia of *Penicillium rubens* that were formed at lower *a*_*w*_ was investigated, to verify whether endogenous polyol reserves can be used to attract water. Dynamic Vapor Sorption (DVS) a method to gravimetrically monitor a sample's water uptake under controlled humidity conditions, was used for this. Secondly, the effect of *a*_*w*_ during sporulation on hyphal growth rates in mycelium originating from such conidia was investigated on both agar and gypsum substrates. This way, a new mechanism through which solute reserves of stressed conidia might contribute to fungal growth is explored.

## 2 EXPERIMENTAL PROCEDURES

### 2.1 Preparation of culture medium with adjusted *a*_*w*_

Plates of Malt Extract Agar (MEA) with modified *a*_*w*_ were prepared from an autoclaved mixture of 25 g MEA (Oxoid) and 500 ml of aqueous NaCl (Merck) solution. The *a*_*w*_ of the NaCl solution was controlled through the concentration of NaCl according to Chen (1989). An *a*_*w*_‐meter (Labtouch‐aw Basic, Novasina) was used to determine the *a*_*w*_ of the agar plates after solidification.

### 2.2 Preconditioning and harvesting of conidia

MEA plates with adjusted *a*_w_ were inoculated by spreading 0.1 ml of stock conidial suspension of *Penicillium rubens* (strain CBS 401.92; CBS Fungal Biodiversity Centre, Utrecht, the Netherlands) with a concentration of 7 × 10^6^ conidia·ml^−1^ over the plate with a spatula. The inoculated plates were incubated at 23°C until sporulation. By subsequently harvesting conidia from these plates, conidia developed at a controlled *a*_*w*_ were obtained. The *a*_*w*_ of the plates used for harvesting was 0.86 ± 0.005, 0.89 ± 0.005, 0.91 ± 0.005, 0.93 ± 0.005, 0.95 ± 0.005, or 0.98 ± 0.005. No sporulating colonies could be grown on plates with an *a*_*w*_ below 0.86. Conidia were also harvested from unmodified MEA plates (with *a*_*w *_= 0.99).

Dry, that is., unwashed conidia where harvested from well sporulating colonies on the *a*_*w*_‐adjusted plates by rolling 0.71–1.00 mm sized glass beads (Sigma Aldrich) over the colony surface with a pair of tweezers. Inspection with a microscope revealed that conidia stuck to the surface of the glass beads, while no conidiophores or hyphal fragments were dragged along. To estimate the number of conidia present on a single glass bead, some glass beads with conidia were put in a volume of water, which was then vortexed to bring the conidia in suspension. Subsequently, the concentration of conidia was determined with a hemocytometer. This way, it was determined that the number of conidia on a single bead was in the order of 6 ± 2 × 10^4^ for conidia formed at any *a*_*w*_.

As an alternative method for harvesting, a conidial suspension was harvested. This was done by wetting sporulating colonies on *a*_*w*_‐adjusted plates with sterile distilled water (SDW) that was supplemented with 0.05% Tween80, and then gently scraping the surface of the colonies. The conidial suspension was then filtered with sterile glass wool to remove mycelium fragments. Subsequently, the suspension was twice pelleted by centrifugation, followed by resuspending in SDW with 0.05% Tween80. Then the suspension was pelleted a third time and resuspended in SDW. The concentration of the suspension was determined with a hemocytometer and then adjusted to a concentration of 10^6^ conidia·ml^−1^.

### 2.3 Ion chromatography

Ion chromatography was used to investigate how much additional NaCl was carried by dry‐harvested, pretreated conidia. To this end, 10 beads with dry‐harvested, pretreated conidia grown at *a*_w _= 0.91 were put in SDW in an Eppendorf tube. The same was done for dry‐harvested conidia grown on MEA (*a*_*w *_= 0.99). The tubes were vortexed to bring the conidia on the beads in suspension. The concentration of conidia in the suspension was then determined with a hemocytometer. Subsequently, the tubes were centrifuged to remove beads and conidia from the fluid. Next, the concentration of Na^+^ ions of the fluid was tested with an Ion Chromatograph (ICS‐90, Dionex). The Na^+^ concentrations and concentrations of the conidial suspensions showed that conidia from MEA released approximately 0.5 pg NaCl per conidium to the fluid, whereas conidia formed at *a*_*w *_= 0.91 released approximately 5 pg NaCl per conidium.

### 2.4 Dynamic vapor sorption measurements on dry conidia

A dynamic vapor sorption (DVS) apparatus (Q5000SA, TA instruments) was used to find the effect of the *a*_*w*_ pre‐treatment on the water vapor sorption of conidia. DVS gravimetrically determines (with a precision of 0.1 μg) the change in water content of a sample while exposing it to a controlled *RH*.

For each measurement, 30 glass beads with dry‐harvested conidia were used as sample for the DVS. Prior to harvesting the conidia, the 30 clean glass beads were weighed with the DVS, so that the total weight of harvested conidia could be determined later on. After each measurement, the 30 beads with conidia were put in SDW to put the conidia in suspension and the concentration was counted with a hemocytometer to determine the actual number of conidia that were present on the beads during the measurement.

During each measurement, conidia were exposed to a set of different *RH*s. The conidia were initially equilibrated with *RH *= 60%. The *RH* was subsequently changed to 67%, 74%, 76%, 78%, 80%, 82%, 84%, 86%, 88%, 90%, 92%, 94%, 96%, and 98%. Next, the same set of *RH*s was applied in reverse order and the *RH* then continued down to 50%, 40%, 30%, 20%, 10%, and 0%. Finally, the complete sequence from 0% back to 98% was repeated once more. After each *RH* step, conidia either attracted or relinquished water, which was gravimetrically monitored by the DVS. A new step in *RH* was initiated only after the sample weight stabilized, that is, when its weight had not changed more than 0.01% during the last 30 min of exposure.

### 2.5 Dynamic vapor sorption measurements on washed conidia

As a control, the sorption behavior of washed conidia was measured with DVS. Conidial suspension from a colony grown at *a*_*w *_= 0.91, prepared as described above, was pelleted through centrifugation once more, after which conidia were harvested from the pellet by rolling 30 glass beads over it. Other than this modification of the harvesting protocol, the same protocol for DVS measurements as described above was followed.

### 2.6 Hyphal growth rates on gypsum

Hyphal growth rates originating from pretreated conidia on gypsum were determined with video microscopy (van Laarhoven et al., 2015). Colored gypsum substrates with added Czapek were inoculated by pipetting 5 μl of conidial suspension onto them. Samples were then dried for 20 min on an analytic balance (Mettler Toledo) until the fluid from the suspension had evaporated. Subsequently, the inoculated samples were incubated in sealed containers in which the *RH* was controlled with aqueous glycerol solutions of defined *a*_*w*_ (Forney & Brandl, 1992). Samples were incubated at an *RH* of either 97% or 90%. After incubation, an image of the sample surface was taken every hour with a digital microscope (7013MZT, Dino‐Lite, numeric aperture 0.22, optical resolution ~1.5 μm). This way, movies of hyphal growth were recorded. The hyphal growth rates were determined from these images with a custom MATLAB script.

### 2.7 Colony diameter extension rates on agar

In addition, average hyphal growth rates originating from pretreated conidia on agar were determined by measuring colony diameter as a function of time. MEA plates with an adjusted *a*_*w*_ of either 0.90 or 0.99 were inoculated with dry‐harvested, pretreated conidia by putting a single glass bead with conidia at the center of the plates. Alternatively, the plates were point‐inoculated with washed, pretreated conidia by pipetting 5 μl of conidial suspension at the center of the plate. The inoculated plates were then incubated at 23°C. To quantify colony extension rates, photographs of the colonies were taken every 2 days with digital camera (Nikon). The size of the colonies as a function of time and, subsequently, the colony extension rates were determined from the images with MATLAB as reported by Bekker (2014).

### 2.8 Dry conidia on microscope slides

To inspect the water sorption of pretreated conidia visually, conidia were dry‐harvested from well sporulating colonies by pressing a clean microscope slide onto the colony. Prior to harvesting, the microscope slides were cleaned with demiwater and isopropanol. Subsequently, the microscopy slides with pretreated conidia were incubated in transparent containers with a *RH* of either 51%, 89% or 96%, as controlled by glycerol solutions at the bottom. 3 hr after incubation, conidia were inspected with a brightfield microscope (Zeiss).

## 3 RESULTS AND DISCUSSION

### 3.1 Water sorption of pretreated conidia

**Figure 1:**
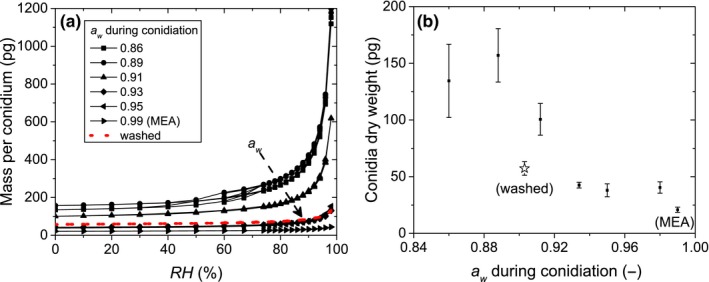
(a) sorption behavior of dry‐harvested conidia formed at different *a*_*w*_. The sorption isotherm data show the combined weight of a single conidium and the water it attracts as a function of *RH*. The arrow marks the direction of the *a*_*w*_ during conidiation. The red, dotted line gives the sorption isotherm that was measured for washed conidia that were formed at *a*_*w *_= 0.91. (b) The weight of dry‐harvested conidia that equilibrated with *RH *= 0%, that is, the dry weight, as a function of the *a*_*w*_ at which they were formed. The star‐shaped data point represents the dry weight of a washed conidia formed at lower *a*_*w*_

DVS was used to investigate how the water sorption of conidia is affected by the *a*_*w*_ during conidiation. Figure 1a shows the sorption isotherms of conidia formed under *a*_*w*_ ranging from 0.99 to 0.86. These describe the weight of conidia and the water they attract while equilibrating with a certain *RH*, wherein the increase in weight indicates the adsorption of more water. Each individual sorption isotherm has a typical type III shape (Brunauer, Deming, Edwards Deming, & Teller, 1940), meaning the weight increases monotonously and largely exponentially with increasing Relative Humidity (*RH*). Note that, while both adsorption and desorption curves are shown, hysteresis effects are negligible. During a measurement, when conidia are in equilibrium with *RH *= 0%, the weight measured by the DVS represents the ‘dry’ weight of the conidia. Two general trends in the data can be distinguished:

Firstly, the conidial dry weight increases with decreasing sporulation *a*_*w*_. The dry weight data obtained from the sorption isotherms are plotted as a function of the pretreatment a_w_ in Figure 1b. It is observed that once the pretreatment *a*_*w*_ lies below 0.93, the weight of the formed conidia increases. Approximately, there is a factor 4 difference between the lightest conidia formed at *a*_*w *_= 0.99 and the heaviest conidia, formed at *a*_*w *_= 0.89.

Secondly, it can be seen that conidia formed at lower *a*_*w*_ have a higher water uptake. Comparing the conidial dry weight to the maximum weight obtained at *RH *= 98%, it is seen that conidia formed at *a*_*w *_= 0.86 and *a*_*w *_= 0.89 attract around seven times their own weight in water, conidia formed at *a*_*w *_= 0.91 attract about five times their weight in water, conidia formed at *a*_*w *_= 0.93 and *a*_*w *_= 0.95 attract around three times their weight in water, and conidia formed at *a*_*w *_= 0.99 attract around once their weight in water.

The sorption behavior of washed conidia that were formed at *a*_*w *_= 0.903 was measured as well, and is represented by the red, dotted line and star‐shaped data point in Figure 1a and b, respectively. It can be seen that washing the conidia results in a marked decrease in the conidial dry weight and a reduction in the sorption capacity as compared to dry‐harvested conidia formed at *a*_*w *_= 0.912 or lower.

**Figure 2:**
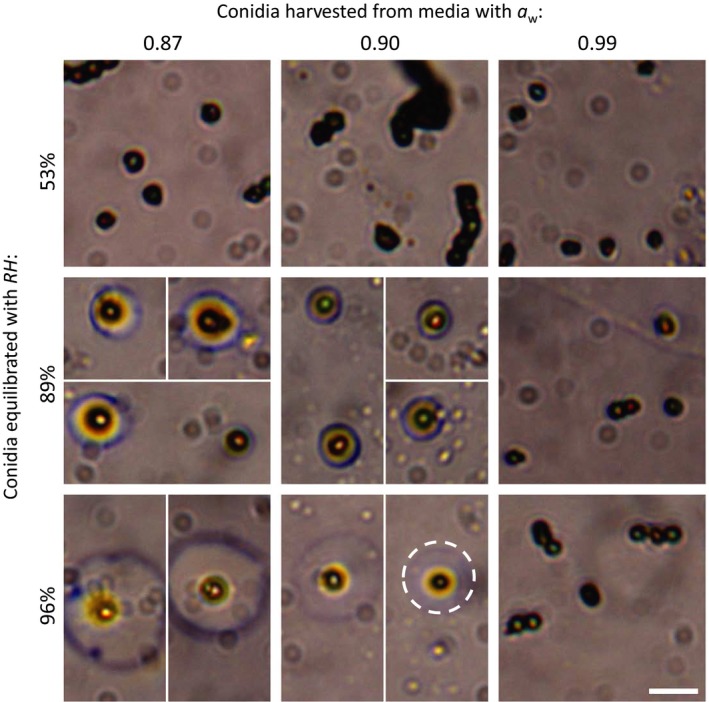
Images of dry‐harvested conidia on microscope slides that are equilibrated with a certain *RH* (51%, 89%, or 96%). The conidia were harvested from either regular MEA (*a*_*w *_= 0.99) or MEA with NaCl added to set the *a*_*w*_ to 0.90 or 0.87. At *RH*s of 89% and 96%, conidia from *a*_*w *_= 0.87 or *a*_*w *_= 0.90 have attracted a visible amount of water, as indicated by the dashed circle. The scale bar represents 10 μm

Figure 1a shows that conidia formed at *a*_*w *_≤ 0.89 attract over 1 ng of water. This corresponds to a volume of 1 pl, which is more than 15 times larger than the typical volume of the conidia of *P. rubens*. Freshly formed conidia have a typical diameter of 3 μm, or a volume of approximately 0.015 pl. Later, during the swelling stages of germination, *P. rubens’* conidia may increase to typically 5 μm in diameter, that is, to a volume of approximately 0.065 pl (Bekker et al., 2012; Samson, Houbraken, Thrane, Frisvad, & Andersen, 2010). It is therefore very unlikely a conidium absorbs 1 pl water into its interior. Conidia in equilibrium with a controlled *RH* were monitored with a microscope to see whether all water was absorbed. Figure 2 shows images of conidia on glass slides in equilibrium with different *RH*s. It can be seen that conidia formed at *a*_*w *_= 0.87 or *a*_*w *_= 0.90 lie in a small water droplet when in equilibrium with *RH *= 89% and *RH *= 96%,. Moreover, the droplets appear to be bigger for higher *RH*. For *RH *= 50% and for conidia grown at *a*_*w *_= 0.99, no such water droplets were observed. This suggests that the attracted water measured with the DVS (Figure 1) is for a large part located outside the conidia.

### 3.2 The role of NaCl in the experiments

NaCl is a known Hygroscopic salt (Chen, 1989). Experiments were performed to exclude the possibility that the NaCl used to adjust the *a*_*w*_ of the culture medium became attached to the harvested conidia, thereby influencing their sorption behavior. Ion chromatography of dry‐harvested conidia showed that a conidium grown on culture medium with NaCl will release approximately 4.5 pg additional NaCl as compared to a conidium grown on regular MEA.

**Figure 3:**
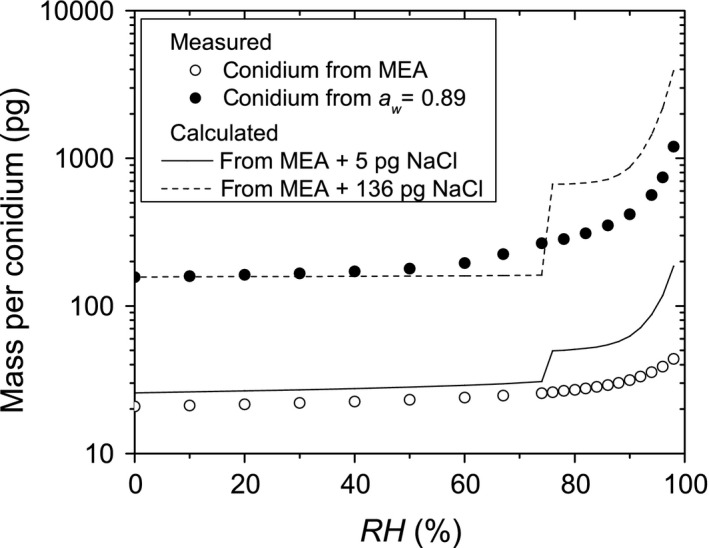
A simple model describing how the sorption of conidia formed on MEA (○) would be modified by the addition of 5 pg NaCl (—) or 136 pg NaCl (^__^). The result is compared to the measured sorption of conidia formed at *a*_*w *_= 0.89 (●)

Figure 3 illustrates the potential impact of 5 pg additional NaCl on the sorption isotherm of conidia grown on MEA. The theoretical sorption curve of a conidium with 5 pg NaCl was calculated as the sum of the sorption of a conidium from MEA (Figure 1a) and the sorption of 5 pg NaCl (Chen, 1989). When this is compared to the sorption of conidia grown at *a*_*w *_= 0.89, two marked differences show. Firstly, the sorption of a conidium from MEA with the additional 5 pg NaCl is much lower than sorption of a conidium from *a*_*w *_= 0.89. Secondly, sorption of the conidium with NaCl has a distinctive and stepwise jump around RH = 75%. The latter phenomenon is caused by the fact that NaCl solutions are saturated at *a*_*w*_=0.75, the deliquescence point of NaCl (Dupas‐Langlet, Benali, Pezron, Saleh, & Metlas‐Komunjer, 2013). Once the *RH* becomes lower than 75%, all solvent water will therefore evaporate while the NaCl crystallizes, which causes the step change in sorption. The deliquescence point would show even in a conidium containing 136 pg of NaCl, 136 pg being the difference in dry mass of conidia from MEA and from *a*_*w *_= 0.89. We therefore conclude that the enhanced water sorption of conidia formed at lower *a*_*w*_ is not caused by NaCl from the culture medium.

While NaCl has been used by other researchers as an osmolytes to tune the *a*_*w*_ of culture medium (Deacon, 2006; Parra, Aldred, Archer, & Magan, 2004), it is generally considered to be inhibitory when compared with other, more popular osmolytes such as glycerol (e.g., Judet et al., 2008; Ponizovskaya et al., 2011; Sautour et al., 2000). The reason to use NaCl as the osmolyte rather than glycerol was twofold. Firstly, its contribution to the sorption behavior could be easily isolated with ion chromatography as described above. Secondly, for *P. rubens* specifically and many other fungi in general, the production of glycerol as a compatible solute plays an important role in the response to water (Blomberg & Adler, 1992; Luard, 1982) and might even play a role as an extra carbon source (Patriarca, Larumbe, Buerra, & Vaamonde, 2011). This makes glycerol a less suitable osmolyte in experiments aimed at probing the response to water stress.

### 3.3 Origin of the additional water sorption by conidia formed at low a_w_

The water droplets around the conidia at equilibrium with the higher *RH*s, as shown in Figure 2, can only exist when the *a*_*w*_ of the droplets matches those *RH*s. This suggests that a substantial concentration of solutes is present in the droplets. This is also suggested by the shape of the measured sorption isotherms in Figure 1. The absence of hysteresis and step‐wise changes point to the formation of aqueous solutions through sorption (Andrade, Lemus & Perez, et al., 2011).

**Figure 4:**
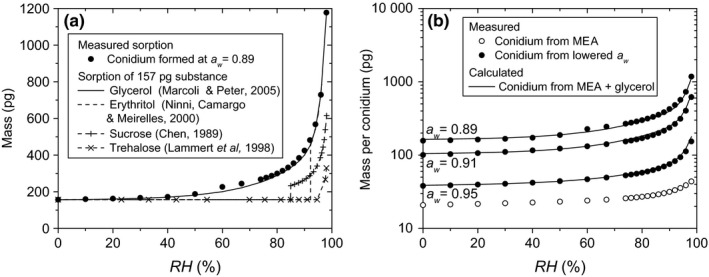
(a) comparison of the sorption behavior of several compatible solutes (lines) as compared to the measured sorption of a conidium formed at *a*_*w *_= 0.89 (●). For each polyol, the relation between concentration and *a*_*w*_ of its aqueous solution is used to plot the mass of the solution as a function of *RH* when the amount of solute is kept constant. Each time, an amount of polyol corresponding to the dry mass of a conidium was chosen to visually compare the sorption of the solutions to that of the conidium. (b) a simple model describing how the sorption of conidia formed on MEA (○) would be modified by different amounts of glycerol. The result is compared to the measured sorption of conidia formed at lowered *a*_*w*_ (●). The mass of the glycerol is chosen to be equal to the difference in dry weight between conidia formed at lowered *a*_*w*_ and those formed on MEA. The modeled sorption (^_^) is defined as the sum of the sorption of the glycerol solution and that of the conidia formed on MEA

Since it is known that conidia formed at lowered *a*_*w*_ carry higher amounts of polyols and sugars (Chandler et al., 2005; Hallsworth & Magan, 1995; Magan, 2006; Nesci et al., 2004), the solutes in the attracted water might be such polyols or sugars, released from the conidia once they are exposed to humid air. To explore which compatible solutes may cause the observed increase in water attraction, the sorption of the typical compatible solutes of *P. rubens*, glycerol, erythritol, sucrose, and trehalose (Luard, 1982; Magan, 2006) is compared to the enhanced sorption of a conidium formed at *a*_*w*_=0.89 (Figure 4a). For each solute, the sorption of an amount of that solute with a mass equal to the dry mass of the conidium is shown. Of these examples, only the sorption isotherm of glycerol has a shape similar to the sorption isotherm of the conidia; trehalose, sucrose and erythritol each display deliquescence at high *RH* (Dupas‐Langlet et al., 2013; Embuscado & Patil, 2001; Lammert, Schmidt, & Day, 1998), similar to NaCl. Moreover, the amount of water sorption of the glycerol matches the sorption of the conidium well.

To further investigate whether glycerol may explain the increased sorption of conidia formed at lower *a*_*w*_, the sorption of conidia with additional glycerol reserves were approximated analogously to the approach shown in Figure 3. Sorption of conidia formed at lower *a*_*w*_ was modeled as the sum of the sorption isotherms of a quantity of glycerol and of conidia formed on MEA. The quantity of glycerol was chosen equal to the difference between the dry weight of conidia from MEA and that of conidia from MEA with lower *a*_*w*_. The approximated and measured sorption isotherms are shown in Figure 4b, showing an excellent match.

This analysis indicates that the water sorption behavior of conidia of *P. rubens* formed at lower *a*_*w*_ may be related to compatible solutes stored during sporulation. Glycerol is a likely candidate, as it is the main compatible solute for *Penicilium* (Luard, 1982) and because the sorption isotherms of glycerol and conidia in Figure 4 match perfectly. The question arises where these reserves are located: in or on the conidia, or both? The data presented here indicate that at least part of the reserves is located on the conidia. Conidia formed at *a*_*w *_= 0.86 have a dry mass of about 140 pg, exceeding the dry mass of conidia formed at *a*_*w *_= 0.99 with around 100 pg. 100 pg of glycerol has a volume of about 80 μm^3^. The microscopy data (Figure 2) showed that *dry‐harvested* conidia formed on MEA have a diameter of around 3.5 μm, or an approximate volume of 20 μm^3^, whereas *dry‐harvested* conidia formed at *a*_*w *_= 0.86 have a diameter of around 4.3 μm, or an approximate volume of around 40 μm^3^. It is therefore unlikely that conidia formed at *a*_*w *_= 0.86 contain 100 pg of glycerol intracellularly. Rather, at least part of the solutes stored in conidia formed at lowered *a*_*w*_ seems to be attached to the conidia exterior. The fact that washing the conidia reduces both their dry weight and their sorption capabilities (1) further implies that extracellularly stored solutes are involved.

Little information is available on possible exterior storage of solutes on the exterior of newly formed conidia. The formation of a water soluble matrix of glycoproteins and polysaccharides during conidiogenesis has been reported for plant pathogen fungi that form sporodochia or acervulli (Louis & Cooke, 1983; Montazeri, Greaves, & Magan, 2003). Such a matrix on conidia has, however, never been reported for fungi that produce conidia on simpler conidiophores like *P. rubens*, although conidia of *P. rubens* have been seen to actively produce an extracellular matrix in the early stages of germination (Bekker et al., 2012). The compatible solutes produced and stored by fungi under water stress conditions are typically regarded as endogenous (Chandler et al., 2005; Hallsworth & Magan, 1995; Magan, 2006; Nesci et al., 2004). These researchers each worked with *washed* conidia, which would eliminate the presence of possible extracellular solutes.

Glycerol located in the conidia interior would need to diffuse to the conidia exterior before contributing to the sorption of droplets as shown in Figure 2. For yeast fungi such as *S. cerevisiae*, it has been shown, however, that glycerol is unable to freely migrate through the membrane and requires Fps1p channels to be transported (Oliveira, Lages, Silva‐Graca, & Lucas, 2003; Tamas, Rep, Thevelein, & Hohman, 2000). This suggests that active processes or at least opened protein channels for facilitated diffusion are required for intracellular glycerol to leave the conidia. Several researchers have reported the loss of endogenous solutes from fungal spores when they were washed with liquid of a higher *a*_*w*_ than that of the conidia interior, as opposed to washing with isotonic solutions (Kelly & Budd, 1991; Luard,^1982^; Magan, 2006; Pascual, Melgarejo, & Magan, 2002; Ypsilos & Magan, 2004). This indicates that diffusion of solutes from ungerminated conidia is possible, although the question remains whether this implies active processes in the conidia.

### 3.4 Hyphal growth rates originating from pretreated conidia

To study the effect of *a*_*w*_ during the formation of conidia on hyphal growth rates in the mycelium that grows from those conidia, growth experiments were performed on both agar and gypsum substrates with pretreated conidia.

**Figure 5:**
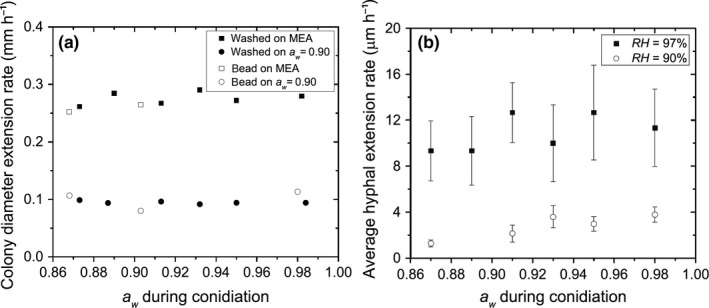
The effect of *a*_*w*_ pretreatment of conidia on subsequent hyphal extension rates. (a) Extension rates of colonies on MEA (squares) or MEA with a lowered *a*_*w*_ of 0.90 (circles). Error bars fall within the symbols (*n* = 3). The agar was inoculated with either conidial suspension (closed symbols) or with dry‐harvested conidia (open symbols). (b) Hyphal extension rates on gypsum equilibrated with vapor of *RH *= 97%(■) or *RH *= 90% (□). The hatched lines represent the standard deviation in measured extension rates (*n* ≥ 15)

In growth experiments on agar, hyphal extension rates were quantified in terms of the radial extension rate of the colony. Experiments were performed with both regular Malt Extract Agar (MEA) and MEA with *a*_*w*_ set to 0.90 through NaCl addition. Figure 5a shows the diameter extension rates of colonies inoculated with conidia formed at different *a*_*w*_. Extension rates were constant from first recorded growth till the end of the experiment, 72 hr later. The data show no clear trend in extension rates with changing pretreatment *a*_*w*_ on either MEA or MEA with *a*_*w *_= 0.90. Moreover, no substantial difference is seen between the extension rates of colonies inoculated with dry conidia (open symbols) and those inoculated with conidial suspension (closed symbols).

In the growth experiments on gypsum, the hyphal extension rates of individual hyphae were obtained with video microscopy. Experiments were performed with both high and low *RH* (97% and 90%). Figure 5b shows the average hyphal extension rates on gypsum samples inoculated with conidia formed at different *a*_*w*_. Extension rates of individual hyphae were constant for as long as they could be recorded. The data show no substantial trend in extension rates with changing pretreatment *a*_*w*_ at either high or low *RH*.

The results show that conidiation *a*_*w*_ does not influence extension rates of *P. rubens* on the long term. This is understandable, as the additional solute reserves in conidia formed under stressful conditions are likely negligible compared to the resource uptake of an established mycelium, even on a substrate as sparse as the gypsum samples equilibrated with *RH*=90%. Other researchers (e.g., Magan, 2006; Dantigny & Nanguy, 2009) did find effects of endogenous reserves on germination time and germ tube extension, which shows that conidiation *a*_*w*_ influences growth only in the earliest stages.

## 4 CONCLUDING REMARKS

In conclusion, we have measured that conidia of *P. rubens* that are formed at a lower *a*_*w*_ have a higher dry weight and attract more water from humid air. This was measured with DVS, which proved to be a powerful tool for approximating these phenomena on the scale of individual conidia. Both phenomena can be explained by conidia originating from lower *a*_*w*_ carrying higher amounts of compatible solutes, that is, glycerol, as indicated by the measured sorption isotherms.

At least part of these solute reserves seems to be located on the conidia exterior, as the observed sorption behavior cannot be explained by endogenous solutes alone. More work is needed to confirm this. Chromatography, spectrometry or electron microscopy techniques could be used to investigate the extracellular presence of high amounts of glycerol in a more direct way. DVS experiments with conidia deactivated by for instance exposure to ethanol vapor (Dantigny, Dao, Dejardin, & Bensoussan, 2007) or NaN_3_ (Bekker et al., 2012) might be performed to investigate whether active release of solutes is required for the enhanced sorption. Growth experiment regarding the influence of sporulation *a*_*w*_ on future growth could be extended by assessing not only the effects on extension rates but those on total biomass formation as well.

The findings of this study might be indicative of important mechanisms through which compatible solute reserves contribute to survival during the early establishment of fungal growth, other than by serving as osmolytes and carbon sources. When extracellularly stored, the solutes might contribute to adhesion. The attraction of additional water likely enhances early foraging for resources and thereby aid germination and early growth. Knowledge of such an extracellular layer of recourses might be important for further studies of (indoor) fungal colonization.

## CONFLICT OF INTEREST

None declared.
